# Astroglia and Tau: New Perspectives

**DOI:** 10.3389/fnagi.2020.00096

**Published:** 2020-04-09

**Authors:** Gabor G. Kovacs

**Affiliations:** ^1^Tanz Centre for Research in Neurodegenerative Disease, University of Toronto, Toronto, ON, Canada; ^2^Department of Laboratory Medicine and Pathobiology, University of Toronto, Toronto, ON, Canada; ^3^Laboratory Medicine Program, University Health Network, Toronto, ON, Canada

**Keywords:** ARTAG, astroglia, astrogliopathy, neurodegeneration, proteinopathy, propagation, tau

## Abstract

Astrocytes contribute to the pathogenesis of neurodegenerative proteinopathies as influencing neuronal degeneration or neuroprotection, and also act as potential mediators of the propagation or elimination of disease-associated proteins. Protein astrogliopathies can be observed in different forms of neurodegenerative conditions. Morphological characterization of astrogliopathy is used only for the classification of tauopathies. Currently, at least six types of astrocytic tau pathologies are distinguished. Astrocytic plaques (AP), tufted astrocytes (TAs), ramified astrocytes (RA), and globular astroglial inclusions are seen predominantly in primary tauopathies, while thorn-shaped astrocytes (TSA) and granular/fuzzy astrocytes (GFA) are evaluated in aging-related tau astrogliopathy (ARTAG). ARTAG can be seen in the white and gray matter and subpial, subependymal, and perivascular locations. Some of these overlap with the features of tau pathology seen in Chronic traumatic encephalopathy (CTE). Furthermore, gray matter ARTAG shares features with primary tauopathy-related astrocytic tau pathology. Sequential distribution patterns have been described for tau astrogliopathies. Importantly, astrocytic tau pathology in primary tauopathies can be observed in brain areas without neuronal tau deposition. The various morphologies of tau astrogliopathy might reflect a role in the propagation of pathological tau protein, an early response to a yet unidentified neurodegeneration-inducing event, or, particularly for ARTAG, a response to a repeated or prolonged pathogenic process such as blood-brain barrier dysfunction or local mechanical impact. The concept of tau astrogliopathies and ARTAG facilitated communication among research disciplines and triggered the investigation of the significance of astrocytic lesions in neurodegenerative conditions.

## Introduction: Concepts of Neurodegenerative Diseases

Neurodegenerative diseases (NDDs) are characterized by progressive dysfunction of neuronal networks. Recent studies have emphasized the role of the supporting tissue, which in the central nervous system comprises astroglia, microglia, and oligodendroglia. Glial responses characterize neuroinflammatory processes and have consequences in the maintenance of the functionality and ion-homeostasis of neurons, and conduction of nerve impulses (Heneka et al., 2014; Ettle et al., 2016; Ferrer, 2017; Verkhratsky et al., 2017). A central aspect of NDDs is the deposition of physicochemically altered variants of physiological proteins in the nervous system (Kovacs, 2019a). Molecular biological, biochemical, genetic and morphological studies identified a spectrum of immunohistochemically and biochemically detectable proteins, which serve as a basis for protein-based disease classification (Kovacs, 2016). Importantly, these pathological proteins accumulate in neurons, astrocytes, and oligodendrocytes (Kovacs et al., 2017a). The microtubule-associated protein tau is one of many proteins that accumulate in pathological forms in NDDs. Additional abnormally deposited proteins include Amyloid-β (Aβ), which is cleaved from the transmembrane amyloid precursor protein (APP), α-Synuclein, Prion protein, Transactive response (TAR) DNA-binding protein 43 (TDP-43), and FET proteins, comprising the fused in sarcoma (FUS), Ewing’s sarcoma RNA-binding protein 1 (EWSR1), and TATA-binding protein-associated factor 15 (TAF15). Other proteins are associated with hereditary disorders encoded by genes linked to neurological trinucleotide repeat disorders, neuroserpin, ferritin-related NDDs, and familial cerebral amyloidoses (Kovacs, 2016). Importantly, in addition to the hallmark pathological lesions of NDDs, combinations of pathological alterations can be observed in the same brain, described as concomitant proteinopathies (comorbidities) with or without additional non-neurodegenerative conditions (i.e., metabolic or vascular), designated as multimorbidities (Kovacs et al., 2008; Rahimi and Kovacs, 2014; Kovacs, 2019b). Therefore, analyses of glial responses should be considered in the context of comorbidities and multimorbidities.

Due to their crucial neuron-supporter role, astroglia reacts to the classical pathogenic events associated with NDDs, such as metabolic changes, molecular damage, and dysregulation of energetic and ion homeostasis (von Bernhardi and Eugenin, 2012). Since protein accumulation is a central event for NDDs, the relationship of astroglia and protein processing systems including the ubiquitin-proteasome system and the autophagy-lysosome pathway (Nijholt et al., 2011) and the unfolded protein response (Cornejo and Hetz, 2013) is important in disease mechanisms. Indeed, the novel concept, referred to as prion-like spreading, suggesting that the proteins associated with NDDs propagate in the nervous system (Brettschneider et al., 2015), have turned scientific interest to the role of glia in the propagation of pathological proteins. The prion-like concept stems from prion disease research, which proposes a template-directed protein misfolding of pathological proteins (Brettschneider et al., 2015). A critical aspect when discussing the role of glia in the processing of pathological proteins is that most of the NDD-related proteins are not expressed by glia in their physiological form. The cell-to-cell propagation theory and the recognition of sequential involvement of anatomical regions led to the description of stages and phases of pathological protein deposits (Kovacs, 2019a). Currently, however, these staging schemes have primarily focused on neuronal (e.g., α-synuclein in Lewy body disorders or tau for neurofibrillary tangle (NFT) formation in Alzheimer’s disease, AD) or extracellular protein deposits (e.g., Aβ in AD), without consideration of glial involvement (Liddelow and Sofroniew, 2019).

To understand the complex interaction of the tau protein and astroglia in NDDs the following points require consideration: (1) alterations of tau protein in disease; (2) which cell types accumulate pathological tau in different disorders; (3) how pathological tau is distributed in the human brain; (4) whether astroglia contributes to the propagation of pathological tau; and finally (5) whether the accumulation of tau in astroglia influence the degeneration of neurons or cause alterations in the physiological neuron-supporting roles of the astroglia.

## Fundamentals of Tauopathies With Relevance to Astroglia

Tauopathies are biochemically, morphologically, and genetically heterogenous NDDs characterized by the accumulation of pathological forms of tau protein in diverse anatomical distribution patterns. Conditions where tau pathology is the driving force in the pathogenesis are called primary tauopathies. They are grouped based on the ratio of 3repeat (R)- and 4R-tau isoforms and two or three major bands (60, 64, and 68 kDa) in the Western blot of sarkosyl-insoluble fractions, the distinct involvement of anatomical areas and cell types affected, and ultrastructural features of tau filaments (Kovacs, 2017; Forrest et al., 2019b). Mutations in the microtubule-associated tau (MAPT) gene are associated with various tau pathologies, which recapitulate features of sporadic tauopathies in the majority of cases (Ghetti et al., 2015; Forrest et al., 2018).

### Biochemical Aspects

Tau protein undergoes several post-translational modifications such as oxidation, glycation, deamination, isomerization, or enzymatically through phosphorylation, methylation, acetylation, O-GlcNAcylation, nitration, proteolytic cleavage (truncation), sumoylation, and ubiquitination (Spillantini and Goedert, 2013; Rösler et al., 2019). These modifications are major driving forces for neurodegeneration, however, tau hyperphosphorylation is thought to be the most important. This potentially leads to altered binding affinity and then to microtubular malfunction with a loss of function mechanism.

Importantly, post-translational modifications of tau are examined in human brains by preparing homogenates of brain tissue that contain different cell types. To evaluate cell-specific modifications, however, mostly immunohistochemical methods are applied. According to a comprehensive study on glial tau pathologies, tau in astrocytes is hyperphosphorylated at different sites including Thr181, Ser199, Ser202-Thr205, Thr212/Ser214, Thr231, Ser262, Ser396-Ser404, and Ser422 (Ferrer et al., 2014). Antibodies Alz50 (amino acids 5–15) and MC-1 (amino acids 312–322) tau revealed an altered conformation of tau in astroglia in tauopathies, but most of the phosphorylated-tau-positive astrocytes are not stained with the antibody tau-C3, which recognizes tau truncated at aspartic acid 421 (Ferrer et al., 2014). Application of antibodies against the carboxy-terminal, middle segment, and amino-terminal of tau show important differences between astroglial tau pathologies [i.e., astrocytic plaques (AP), tufted and ramified astrocytes (RA)] suggesting different truncation patterns (Ferrer et al., 2014, 2018; Ferrer, 2018). Interestingly, astrocytes accumulate predominantly the 4R tau isoform and 3R immunoreactivity is rarely observed (Ferrer et al., 2014). In summary, for an understanding of the role of astroglia in pathological processes involving tau, the application of different tau-antibodies in scientific reports must be carefully considered as this may lead to distinct interpretations.

### Neuropathological Phenotypes

Currently, the nomenclature of primary tauopathies overlaps with that of frontotemporal lobar degeneration (FTLD), and are referred to FTLD-tau (Forrest et al., 2019b). The terminology of clinicopathological phenotypes refers to clinical symptoms, such as progressive supranuclear palsy (PSP), to anatomical distribution of neuronal degeneration, such as corticobasal degeneration (CBD); to morphological features, such as argyrophilic grain disease (AGD) and globular glial tauopathies (GGT); to both clinical and morphological features, such as NFT predominant dementia, which is now included in the spectrum of primary age-related tauopathy (PART); and to a physician referred to as Pick’s disease (PiD). Except for PART, all primary tauopathies accumulate pathological tau in astrocytes. However, in the diagnostic neuropathological practice, astrocytic tau pathology is considered for the distinction of PSP and CBD, and GGT.

Neuropathological definitions of primary tauopathies can be summarized as follows (Kovacs, 2017): (1) Neuronal cytoplasmic spherical Pick bodies that are 3R-tau immunoreactive characterize PiD; (2) PSP is a 4R tauopathy with NFTs in hallmark subcortical structures including the subthalamic nucleus, basal ganglia and brainstem, and additionally tufted astrocytes (TAs), oligodendroglial coiled bodies, and threads; (3) CBD is a 4R tauopathy with diffuse neuronal cytoplasmic immunoreactivity and spherical neuronal inclusions, threads in the white and gray matter, coiled bodies and astrocytic plaques (AP); (4) Neuropathology of AGD include argyrophilic and 4R-tau immunoreactive grains in medial temporal lobe structures that are variably associated with pretangles, oligodendroglial coiled bodies, and astrocytic tau immunoreactivity; (5) Argyrophilic (Gallyas) and 4R-tau immunoreactive globular oligodendroglial and non-argyrophilic (Gallyas), 4R-tau immunoreactive globular astroglial inclusions (GOI and GAI, respectively) characterize three types of GGTs; and (6) Histopathological features of the combined 3R and 4R tauopathy, PART, include NFTs restricted to the hippocampus and medial temporal lobe. Tau morphologies in PART are similar to those observed in AD except for the absence or infrequent Aβ plaques (Crary et al., 2014).

Further peculiar tau pathology in the aging brain is aging-related tau astrogliopathy (ARTAG), which describes tau-positive astroglia accumulating 4R isoform of tau in subpial, subependymal, and perivascular locations, and the white and gray matter (Kovacs et al., 2016). Chronic traumatic encephalopathy (CTE) is the neuropathological term describing mixed 3R- and 4R-tau pathology primarily in neurons with additional astroglial tau morphologies following mild repetitive head injury (McKee et al., 2016). Mostly neuronal 3R- and 4R-tau pathology is associated with an autoimmune process anti-IgLON5 (IgLON family member 5)-related encephalopathy that predominantly affects subcortical structures (Gelpi et al., 2016). Several conditions, including post-infectious, metabolic, and non-*MAPT* gene-related hereditary disorders are associated with tau pathologies primarily affecting neurons (Kovacs, 2017).

### Highlights of Pathogenesis

As a microtubule-associated protein, tau functions in promoting the polymerization and assembly of microtubules (Weingarten et al., 1975). Although tau isoforms with 3R and 4R are equally expressed in the adult human brain (Sergeant et al., 1997), neuronal populations show variability in the expression of 3R- and 4R-tau isoforms (Goedert et al., 1989; Buée et al., 2000). Full-length tau protein is primarily located in axons (Trojanowski et al., 1989; Lee et al., 2001) and is also present in nuclear or somatodendritic compartments of neurons (Tashiro et al., 1997). Further studies suggest that oligodendrocytes (Klein et al., 2002) also express tau and that tau can be detected in interstitial fluid (Yamada et al., 2011). Stimulation of neuronal activity induces tau release from healthy, mature cortical neurons and the phosphorylation of extracellular tau appears reduced in comparison with intracellular tau (Pooler et al., 2013). Tau may also play a role in cell signaling (or act as a buffer for cell signaling), and binds to nucleic acids, and may be involved in chromatin remodeling (for review, see Jadhav et al., 2019). In disease conditions, secreted tau is thought to be crucial in the propagation of tau pathology. It has been suggested that tau is secreted within extracellular vesicles as exosomes (Wang et al., 2017) and ectosomes, which are plasma membrane-originating vesicles, and when it accumulates, the exosomal pathway is activated (Dujardin et al., 2014).

Prion-like cell-to-cell spreading is considered in the propagation of protein (including tau) pathology in a range of NDDs (Brettschneider et al., 2015). This implies that the release of pathological tau is followed by uptake in related neurons leading to the formation of aggregates inside the recipient cells following a seeding process (Goedert et al., 2017b; Mudher et al., 2017; Goedert, 2018). Mice transgenic for wild-type human tau inoculated with homogenates from human primary tauopathy brain extracts recapitulate the hallmark lesions of the original inoculated tauopathies, including astroglial tau pathology (Clavaguera et al., 2009). A recent study also described the rapid and distinct cell-type-specific spread of pathological tau following intracerebral injections of CBD brain extracts into young human mutant P301S tau transgenic mice (Boluda et al., 2015). These studies support the concept of tau strains. This terminology has been used in prion disease research to describe the event that disease-associated proteins maintain unique conformations and associate with similar patterns of pathology. Indeed, a study using a cell system to isolate tau strains from five different tauopathies described different sets of strains related to tau diseases (Sanders et al., 2014). Furthermore, a recent study identified distinct tau strain potency between tauopathies in non-transgenic mice (Narasimhan et al., 2017). Studies supporting the concept of strains have been recently complemented by research investigating the structure of tau filaments in diverse conditions with tau pathology, including AD, PiD and CTE (Falcon et al., 2018a,b; Falcon et al., 2019).

Whether astroglial disease-associated tau itself is capable of seeding is difficult to address in human studies since brain homogenates contain a mixture of neuronal and glial (astroglia and oligodendroglia) pathological tau in primary tauopathies. However, recent reports used brain homogenates derived from brain regions showing ARTAG pathology. Accordingly, it was shown that seeding is produced in neurons of the hippocampal complex, astrocytes, oligodendroglia and along fibers of the corpus callosum, fimbria and fornix following inoculation into the hippocampus of wild type using brain homogenates of ARTAG pathology (Ferrer et al., 2018). Thus, the host tau is also relevant for the seeding and spreading of tau pathology (Ferrer et al., 2020b).

In summary, regarding these pathogenic aspects, the question remains how astrocytes come into contact with modifications of tau proteins and whether astrocytes are major players or bystanders in tau-induced neurodegeneration and propagation of tau pathology.

## The Morphological Spectrum of Astrocytes Accumulating Tau

**Figure 1:**
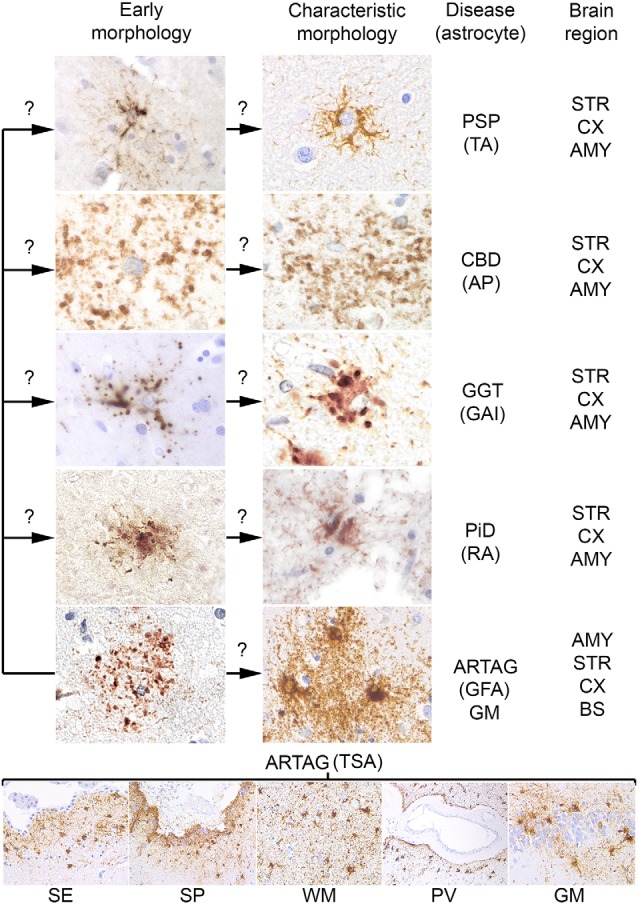
Conceptual summary of astrocytic tau pathologies. Fine granular tau deposition is seen in all primary tauopathies and in gray matter aging-related tau astrogliopathy (ARTAG), which might be interpreted as preceding forms of early morphologies (left column of the upper panel) of disease characteristic morphologies (right column of the upper panel) such as TAs in progressive supranuclear palsy (PSP), astrocytic plaques (AP) in corticobasal degeneration (CBD), globular astroglial inclusions (GAI) in globular glial tauopathies (GGT), ramified astrocytes (RA) in Pick’s disease (PiD) and granular fuzzy astrocytes (GFA) of gray matter ARTAG. Thorn-shaped astrocytes (TSA) are different morphologies detected in subependymal (SE), subpial (SP), white matter (WM), perivascular (PV) location, and rarely in gray matter (GM, here exemplified by the dentate gyrus of the hippocampal formation). Question marks indicate that these aspects are hypothetical. The predominantly affected regions are listed on the right, which does not exclude that other regions show these morphologies. AMY, amygdala; STR, striatum; CX, cortex; BS, brainstem.

For many decades silver stains were the gold standard in neuropathological diagnostic practice. These were used to recognize unusual structures, such as NFTs and inclusion-bodies in degenerating neurons. Although later pathological phenomena have been identified in astrocytes using silver stains (in particular Gallyas staining), their widespread involvement in the pathological process of tauopathies was discovered with the application of immunostaining for phosphorylated tau. Astroglial tau pathologies were described originally as glial fibrillary tangles, by analogy to neuronal NFTs. Later, however, it was recognized that tauopathies associate with dramatically different morphologies. Unfortunately, many studies used different descriptions and terminology when reporting these pathologies, complicating the comparison of studies. However, interpretations still vary between experts calling for consensus studies (Kovacs et al., 2017d). A study aiming to describe aging-related astroglial tau pathologies recommended the distinction of six major morphologies, such as AP, TAs, RA, GAIs, which are seen mostly in primary tauopathies, and thorn-shaped astrocytes (TSA) and granular/fuzzy astrocytes (GFA) observed in ARTAG (Kovacs et al., 2016; Figure 1). It must be emphasized that these are predominating astrocytic inclusions, which distinguish the different tauopathies. However, a variety of additional astroglial morphologies of tau accumulation can be detected in these disorders that do not have, and for the current diagnostic practice not require specific designations. These various morphologies yet to be defined might reflect the different phases of tau aggregation and accumulation in astrocytes whereas the six main morphological types depict the mature and characteristic morphologies.

A further concept that an early event in the development of astrocytic pathology might be the fine granular accumulation of tau in astrocytic processes (Kovacs et al., 2017a,c), which is similar to early pretangle formation in AD, which precedes NFT development (Bancher et al., 1989). This concept suggests that first tau becomes hyperphosphorylated, then misfolds and aggregates, and then forms fibrillar structures. During this process, fine tau deposits are then redistributed to proximal (TAs) or distal (AP) segments of the astrocytic processes, aggregate and become detectable in some silver-stains (Ikeda et al., 2016; Kovacs et al., 2017c; Figure 1).

### Tufted Astrocytes

TAs are hallmark lesions of PSP. Although in 1988, Probst et al. (1988) described stellate neurons showing Gallyas positive material, which could be interpreted today as TAs. The term tufts of abnormal fibers were used first by Hauw et al. (1990) in PSP cases using the Bodian silver method and tau IHC. This was then confirmed to be related to astrocytes (Yamada et al., 1992, 1993). Currently, TAs are defined as astrocytes showing phosphorylated-tau immunoreactivity in the proximal segments of astrocytic processes (Kovacs et al., 2016). TAs are detected in Gallyas and modified Bielschowsky silver staining. Ultrastructural observations describe tubular profiles in these (Nishimura et al., 1992; Arima, 2006). Interestingly, TAs of PSP and AP of CBD seem to accumulate close to blood vessels (Shibuya et al., 2011).

PSP pathology associates with different clinical presentations (Dickson et al., 2010), therefore, the amount and distribution of astrocytic pathology might vary between these forms. Although descriptions of astrocytic tau pathologies vary considerably, rare tuft-shaped astrocytes in the cortex have been reported in cases with a mutation in *MAPT*. These include mutations on exon 1 R5H (Hayashi et al., 2002), TAs in exon 1 R5L mutation (Poorkaj et al., 2002), in intron 9 and 10 *MAPT* mutations (Malkani et al., 2006; Ghetti et al., 2011), and wide range of astrocytic tau morphologies, referred to as tufted forms, have been linked to the deln296 *MAPT* mutation (Ferrer et al., 2003). TAs and AP have also been described in the P301L, P301t, and P301S *MAPT* mutations (Ghetti et al., 2011). Glial tangles and TAs (Stanford et al., 2000) and AP (Halliday et al., 2006) have been described in the S305S mutation.

### Astrocytic Plaques

AP are hallmark lesions of CBD. The observation of tau positive pathology in CBD resembling neuritic plaques without Aβ cores (Mattiace et al., 1991), mainly in the distal parts of astrocytic processes (Feany and Dickson, 1995), opened new avenues for the research on CBD. AP show densely tau-immunoreactive stubby dilatations of distal processes of astrocytes giving a plaque-like appearance (Kovacs et al., 2016), which are argyrophilic on Gallyas silver. Ultrastructurally, AP are characterized by randomly arranged bundles of twisted and straight tubules with diameters of 15–20 nm (Yoshida, 2014). TAs in PSP and AP in CBD are immunoreactive for antibodies detecting the carboxyl-terminal and middle region of tau but do not show immunoreactivity to antibodies detecting the amino-terminal region of tau, suggesting that the latter is reduced or lacking in astrocytic tau pathologies (Ferrer, 2018).

Incidental CBD cases have been reported with astroglial tau pathology in the cortex in the absence of neuronal tau. These findings suggest that tau accumulation in astroglia might represent one of the initial pathological steps in the onset of CBD (Ling et al., 2016). Indeed, CBD pathology can exist in individuals without overt clinical symptoms suggesting that AP are not the major substrates for clinical symptomatology in CBD (Milenkovic and Kovacs, 2013; Martínez-Maldonado et al., 2016), supporting the notion that the regional distribution of neuronal degeneration and tau pathology underlies more the clinical phenotype. In contrast to the predominance of AP in preclinical asymptomatic CBD, neuronal tau aggregates predominated in rapidly progressive fulminant forms of CBD (Ling et al., 2020). In addition to the mutations in *MAPT* associated with TAs in PSP (above), several mutations in *MAPT* are also associated with AP in CBD including the S305S, IVS10+16 and R406W mutations (Forrest et al., 2018).

### Ramified Astrocytes

RA are the characteristic astrocytic lesion in PiD. However, compared to neuronal tau pathology in PiD, the reported density and distribution of RA varies between cases and studies. This is likely attributed to the variability of definitions of PiD before the application of isoform-specific antibodies (Dickson, 1998; Kovacs et al., 2013b). Feany et al. (1996) precisely described that these astrocytic inclusions in PiD “tended to occupy more of the cell body and to ramify into the astrocytic cell processes,” and that they were “often localized to one side of the cell soma.” RA may show 3R-tau immunoreactivity (Kovacs et al., 2013b; Ferrer et al., 2014).

### Globular Astroglial Inclusions

Globular astroglial inclusions are seen in GGT in the gray matter and are defined as tau-immunoreactive globules (1–5 μm) and dots (1–2 μm) in the proximal parts of astrocytic processes and perikarya (Kovacs et al., 2016). These can be reminiscent of TAs in morphology and distribution, but they are non-argyrophilic on Gallyas and Bielschowsky silver stains (Ahmed et al., 2013; Kovacs, 2015). GGT types (I–III) are distinguished based on the anatomical involvement and the predominance of oligodendroglial or astroglial inclusions (Ahmed et al., 2013). Globular astroglial inclusions have been associated with mutations in *MAPT* including IVS10+16, P301L, K317N, and K317M (Zarranz et al., 2005; Tacik et al., 2015, 2017; Forrest et al., 2018, 2019a). Altered expression of several functional markers of astroglia (and oligodendroglia) in GGT supports the notion that these are primary astrogliopathies (and oligodendrogliopathies; Ferrer et al., 2020a) opening new avenues to understand NDDs.

### Thorn-Shaped Astrocytes

Currently, TSAs are discussed in the frame of ARTAG (Kovacs et al., 2016). They are defined as tau-immunoreactivity in astrocytic perikarya with extension into the proximal astrocytic processes, as well as tau-immunopositive inclusions in the astrocytic endfeet at the glia limitans at the pial surface and around blood vessels (Kovacs et al., 2016). TSAs are argyrophilic on Gallyas silver. Nishimura et al. (1992) reported argyrophilic masses with flame- or thorn-like shape in addition to TAs in PSP, which is likely to represent an early description of TSAs. TSAs were also reported in the aging brain in the subpial and subependymal regions, and frequently in the depths of gyri, and the basal forebrain and brainstem (Ikeda et al., 1995, 1998; Ikeda, 1996). Schultz et al. (2000, 2004) reported a high prevalence of TSAs at the level of the amygdala, furthermore, they observed similar astroglial tau pathology in aged baboons. TSAs are immunoreactive for antibodies detecting the carboxyl- and amino-terminals and middle region of tau, show 4Rtau phosphorylation at several specific sites and abnormal tau conformation, but they lack ubiquitin and show increased superoxide dismutase 2 (SOD2) immunoreactivity (Ferrer et al., 2018). Moreover, phosphoproteomics of dissected vulnerable regions identifies an increase of phosphorylation marks in a large number of proteins such as GFAP, aquaporin 4, several serine-threonine kinases, microtubule-associated proteins and other neuronal proteins (Ferrer et al., 2018).

### Granular-Fuzzy Astrocytes

GFA is the second type of astrocytic inclusions associated with ARTAG. GFAs are characterized by fine granular tau-immunoreactivity in processes of gray matter astrocytes with dense tau-immunoreactivity in the perinuclear soma also observed in most astrocytic inclusions (Kovacs et al., 2016). GFAs are largely non-argyrophilic on Gallyas silver-staining, except some of the dense perikaryal accumulations.

In addition to the characteristic tau-immunopositive grains, tau-immunopositive astrocytes have been described in AGD. These are referred to as bush-like astrocytes and have a similar anatomical distribution to GFAs in ARTAG (Botez et al., 1999). Due to the morphological similarities and anatomical overlap of astrocytic inclusions in AGD and ARTAG, the bush-like astrocytes in AGD are better interpreted as GFAs (Kovacs et al., 2016). A study on PSP and AGD indicated that some GFA-like morphologies (termed tufted astrocyte (TA)-like astrocytic lesions in that study) can potentially evolve into Gallyas-positive TAs in AGD brains (Ikeda et al., 2016). GFAs can be seen in a wide range of neurodegenerative conditions, including frontotemporal tauopathies, AD, prion disease and Huntington’s disease, and neurologically normal controls (Kovacs et al., 2017b,c; Baskota et al., 2019).

### Other Morphologies and Combinations

Additional tau-immunoreactive astrocytes are also described in cases with mutations in *MAPT*. However, meticulous descriptions of the morphological and anatomical distributions of tau inclusions in astrocytes are often missing in publications and the lack of concise consensus nomenclature on astroglial tau pathologies does not facilitate clear comparison of these publications. Usually, mutations in *MAPT* on exons 1, 10, 11, and 12, as well as introns following exons 9 and 10, show astroglial inclusions (Ghetti et al., 2011). A peculiar type of astrocytic tau pathology has been described in a familial behavioral variant frontotemporal dementia disorder where no known mutations in *APP, PSEN1*, *PSEN2, MAPT, GRN, TARDBP*, and *FUS* and no pathological expansion in *C9ORF72* have been found (Ferrer et al., 2018). The morphology of astrocytic inclusions were similar to reactive astrocytes. In addition to the cytoplasm, perivascular foot processes around cortical blood vessels, furthermore cerebellar Bergmann glia were also immunostained with the AT8 antibody (double-phosphorylation sites Ser202-Thr205; Ferrer et al., 2015). Widespread TSAs and GFAs have also been described in a novel *GRN* nonsense mutation (c.5G>A: p.Trp2*; Gómez-Tortosa et al., 2019); a mixture of bushy and TAs in *TARDBP* mutation p.Ile383Val (Gelpi et al., 2014); and AP and ARTAG in 17q21.31 duplication (Alexander et al., 2016; Le Guennec et al., 2017).

## What Is the Relevance of ARTAG?

### Types of ARTAG

ARTAG is an umbrella term that includes a spectrum of astrocytic tau morphologies mainly in the aging brain (Kovacs et al., 2016). ARTAG includes morphologies described as TSA and GFA, which can be present in the same brains and anatomical regions together (Kovacs et al., 2016). The two types of tau immunoreactivities are present in different locations defined as types of ARTAG. TSAs are seen mostly in subpial, subependymal, or perivascular areas, as well as in the white matter (WM). TSAs are less frequently observed in the gray matter. In contrast, GFAs are observed only in the gray matter. Accordingly, ARTAG is classified as subependymal, subpial, perivascular, white or gray matter ARTAG. Clustering of TSAs and GFAs can be observed in both the white and gray matter.

### Distribution Patterns of ARTAG

Using conditional probability and logistic regression to model the sequential distribution of ARTAG across different brain regions, a recent study evaluated the frequencies and hierarchical clustering of anatomical involvement of astrocytic inclusions (Kovacs et al., 2018a). Overlapping sequential distribution patterns have been recognized for the different types of ARTAG.

**Figure 2:**
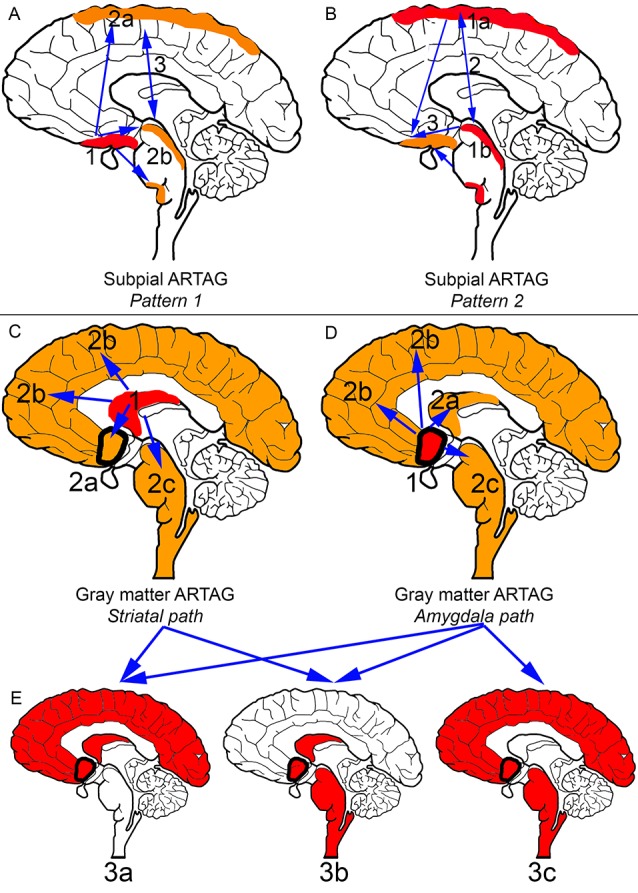
Distribution patterns of subpial and gray matter ARTAG. In all panels, brain regions colored red represent initial or combined regions of astrocytic involvement and brain regions colored orange represent subsequent brain regions involved. Brain regions outlined in a thick black line represent the amygdala. **(A)** Subpial ARTAG, pattern 1. Basal brain regions show subpial ARTAG first (stage 1), which is followed by a bidirectional sequence to rostral (lobar, stage 2a) or caudal (brainstem, stage 2b) regions. Both brain regions are usually affected together (stage 3, indicated by double-headed arrow). **(B)** Subpial ARTAG, pattern 2. Lobar (stage 1a) or brainstem (1b) involvement is followed by the involvement of both brain regions (stage 2), followed by the involvement of basal brain regions (stage 3, indicated by double-headed arrow). **(C)** Gray matter ARTAG, striatal pathway. From the striatum (stage 1), astrocytic tau accumulation proceeds towards the amygdala (stage 2a, blue arrow), or cortex (stage 2b, blue arrow), or rarely to the brainstem (stage 2c, blue arrow). **(D)** Gray matter ARTAG, amygdala pathway. The amygdala (stage 1) precedes the involvement of the striatum (stage 2a, blue arrow), the cortex (stage 2b, blue arrow) or the brainstem (stage 2c, blue arrow). **(E)** Both striatal and amygdala pathway includes stage 3a (striatum + amygdala + cortex), or stage 3b (striatum + amygdala + brainstem), and only the amygdala pathway stage 3c (amygdala +cortex + brainstem), which then eventually involve all regions (stage 4, not indicated).

In the aging brain subpial ARTAG shows two distinct patterns (Figures 2A,B; Kovacs et al., 2018a). Pattern 1 is characterized by the involvement of basal forebrain regions first (stage 1). This is followed by a sequence either rostral (lobar, stage 2a) or caudal (brainstem, stage 2b), which regions are then affected together (stage 3). Pattern 2 starts in lobar regions (stage 1a) or the brainstem (stage 1b), followed by the involvement of both (stage 2) and preceding basal forebrain regions (stage 3). Importantly, subpial astrocytic tau accumulation is prominent in CBD, which must be distinguished from subpial TSAs in aging and other conditions on a morphological level and the distinct distribution pattern (Kovacs et al., 2018a).

Pattern 1 described above for subpial ARTAG is also recognized for WM ARTAG. In the second pattern of WM ARTAG, lobar involvement (stage 1) is followed by basal forebrain regions (stage 2a) or the brainstem (stage 2b), finally evolving into the involvement of all regions (stage 3; Kovacs et al., 2018a).

Gray matter ARTAG (GFA morphology) seems to follow two distinct patterns (Figures 2C–E). One involves the striatal pathway, which is reminiscent of the pattern seen in PSP but lacking further morphological characteristics of PSP (i.e., subcortical NFTs and accumulation of typical TAs). Accordingly, from the striatum (stage 1) astroglial tau in the form of GFAs proceeds to the amygdala (stage 2a), cortex (stage 2b) or rarely to the brainstem (stage 2c). Following stage 3a (striatum and amygdala and cortex), or stage 3b (striatum and amygdala and brainstem), all regions are affected (stage 4; Kovacs et al., 2018a). The constellation of striatum and cortical and brainstem GFA involvement has not been observed, hence the absence of a stage 3c. The second pattern of gray matter ARTAG is referred to as the “amygdala first” pattern where involvement of the amygdala (stage 1) precedes the striatum (stage 2a), the cortex (stage 2b) or the brainstem (stage 2c). This evolves into three combinations of stage 3 (a: amygdala and striatum and cortex; b: amygdala and striatum and brainstem; c: amygdala and cortex and brainstem) and finally all regions are involved (stage 4; Kovacs et al., 2018a).

The sequential pattern of GFA involvement in the gray matter could represent the involvement of astroglia in the propagation of pathological tau. However, for subpial, WM, perivascular, and subependymal, ARTAG, it might reflect the consequences of a repeated or permanent pathogenic process, such as one associated with the blood-brain barrier. This is supported by the observation of increased connexin-43 (gap junction protein in astrocytes) and aquaporin-4 (a marker of astrocytes associated with water transfer) in these astrocytes (Kovacs et al., 2018b). Subpial ARTAG initiated in basal regions (i.e., pattern 1) proceeding towards the convexity of the brain could suggest a disease mechanism related to the circulation of the cerebrospinal fluid (Kovacs et al., 2018a). In contrast, ARTAG initiated in the dorsolateral lobar areas (i.e., pattern 2) and dorsolateral parts of the brainstem suggest a local inducing factor (i.e., mechanical), including in some cases mild traumatic brain injury (Kovacs et al., 2018a).

### Clinical Relevance of ARTAG

Following the examination of patients with a non-fluent variant of primary progressive aphasia associated with AD pathology (logopenic variant of primary progressive aphasia), Munoz et al. (2007) was the first group to discuss the possibility that TSAs may have clinical significance. The authors referred to these astrocytic inclusions as “argyrophilic thorny astrocyte clusters (ATACs)” and described them in the frontal, temporal, and parietal cortices and in the subcortical WM (Munoz et al., 2007). Other studies, however, did not found a clear association between ATACs and focal syndromes (Mesulam et al., 2008; Bigio et al., 2010). Recent studies have shown that lobar WM ARTAG is frequent in AD-related pathology (Kovacs et al., 2017c). A recent study confirmed this and further demonstrated that lobar ARTAG correlates with older age and higher Braak stage. However, ARTAG pathology was found equally prevalent in AD cases with typical and atypical clinical presentations (Nolan et al., 2019). A further interesting aspect of WM ARTAG in AD has been recently highlighted. Accordingly, irrespective of the regional NFT burden worsening language and visuospatial functions were correlated with higher TSA density in language-related and visuospatial-related regions, respectively (Resende et al., 2020).

Widespread distribution of gray matter GFAs has also been reported in elderly patients with cognitive decline with or without parkinsonism (Kovacs et al., 2011). In a subsequent study in an aging cohort, four different patterns of ARTAG were described based on the anatomical distribution of the tau astrogliopathy and its combination with neuronal tau pathology (Kovacs et al., 2013a). These include: (1) medial temporal lobe type; (2) amygdala type; (3) limbic-basal ganglia-nigral type with neuronal tauopathy; and (4) hippocampus-dentate gyrus-amygdala type with neuronal tauopathy (Kovacs et al., 2013a). These different ARTAG patterns overlap with the sequential distribution patterns described above for gray matter ARTAG (Kovacs et al., 2018a). However, TSAs in the dentate gyrus of the hippocampus seems to be an unusual feature, which was recognized by other groups as well (Lace et al., 2012). Indeed, mathematical modeling of hippocampal tau immunolabeling patterns suggested tau astrogliopathy in the elderly involve hippocampal subregions in a different pattern from that observed in primary tauopathies (Milenkovic et al., 2014). The remarkable involvement of the dentate gyrus suggests a distinct pathogenesis, particularly that this subregion might eventually show alterations related to (preclinical?) epileptic mechanisms. A further study involving individuals aged 90 years or older with cognitive decline found an association with cortical ARTAG independent of AD pathology (Robinson et al., 2018).

In summary, depending on the type (e.g., gray matter) and location (e.g., limbic system or WM adjacent to regions related to focal cortical symptoms) of ARTAG pathology it might be a pathological indicator of a reduced threshold, which might lead to decompensation of cognitive functions. Moreover, in combination with other pathologies, even with a different pathogenesis, an additive effect of ARTAG to reach the individual threshold for cognitive decompensation might be seen.

### The Peculiar Relation of ARTAG and CTE

The current neuropathological consensus definition of CTE emphasizes the occurrence of neuronal and astrocytic tau “around small vessels in an irregular pattern at the depths of the cortical sulci” (McKee et al., 2016). However, this definition has been used with various interpretations, which makes many studies difficult to reconcile. Some studies interpret subpial TSAs in the depth of cortical sulci as CTE whereas other studies interpret the definition verbatim, and without neuronal tau did not use the term CTE. Examples of overlapping aspects of astrocytic tau accumulation in brains with CTE and that seen in ARTAG in aging cohorts include accumulation of astrocytes in basal brain regions and dorsolateral lobar areas in subpial, perivascular and gray matter locations, association with ventricular enlargement, or overrepresentation of males (McKee et al., 2013, 2015, 2016; Liu et al., 2016; Kovacs et al., 2017c). Forrest et al. (2019c) assessed the brain regions involved in early CTE stages (i.e., frontal, temporal and parietal cortices) in 310 aged participants in a European community-based population for the presence of CTE and ARTAG. This study found that no cases satisfied current diagnostic criteria for CTE although 117 displayed cortical ARTAG. Isolated phosphorylated-tau pathologies occurring at the depths of cortical sulci were found in 25 individuals (8%; 7 Forrest et al., 2019c), but due to the lack of neuronal tau accumulation in a pathognomonic location (McKee et al., 2016), these isolated features did not warrant the diagnosis of CTE. These observations supported the notion that ARTAG is a common age-related pathology in community populations and CTE is absent or rarely observed (Forrest et al., 2019c). Together, with an additional study, these observations corroborate that isolated phosphorylated-tau immunoreactivities in the correct context could be interpreted only as a possible feature (Bieniek et al., 2020) or component (Forrest et al., 2019c), but in isolation not diagnostic, of CTE pathology.

Today, it is difficult to prove or exclude the possibility that some kinds of traumatic brain injuries (which might be different than those associated with currently defined CTE-related pathology) induce astrocytic tau pathologies in isolation or that astrocytic tau pathologies are the first in some cases of whatever type of trauma. There is a lack of standardized questionnaires on mild and repeated head injuries in population-based studies that have evaluated astrocytic tau pathology systematically, which makes the interpretation of results difficult. Current knowledge suggests that the neuronal tau pathology component is required to diagnose CTE. The generation of a CTE-specific tau antibody could help to reconcile this question. Alternatively, in well-documented cohorts of mild traumatic brain injury, the “components” (Forrest et al., 2019c) or “features” (Bieniek et al., 2020) of CTE pathology could be evaluated in isolation and together. Theoretically, in the same brain, there are likely regions with neuronal tau in the “correct location” for CTE while other regions will show astrocytic pathology in isolation.

### The Relation of ARTAG and Primary Tauopathy-Related Astrogliopathies

TSAs and GFAs are commonly observed in primary tauopathies, although they are not the distinguishing astrocytic inclusions. The concept of the maturation of tau deposition in astrocytes can lead to the appearance of GFA-like morphologies in PSP, CBD, PiD and GGT (Kovacs et al., 2017a,c). The distribution of the striatal type pattern of gray matter ARTAG in the ageing brain is reminiscent of the distribution of astrocytic tau pathology in PSP. Indeed, in PSP striatum (stage 1) is followed by cortical (frontal-parietal to temporal to occipital) involvement (stage 2a and b, respectively) proceeding then to the amygdala (stage 3) and the brainstem (stage 4; Kovacs et al., 2018a). In contrast, for CBD first involves the frontal (including premotor) and parietal cortices (stage 1) proceeding to the temporal and occipital cortices (stage 2). Subsequently and parallelly subcortical areas, including either, or both, the striatum and the amygdala (stage 3) are involved, followed by the brainstem (stage 4) including the midbrain followed rarely by the pons and medulla oblongata.

Moreover, in CBD a distinct morphology, characterized by tau immunoreactivity of subpial astrocytic end-feet, has been recognized. This is the predominant subpial astrocytic tau pathology in CBD independently of subpial ARTAG in basal brain regions (together representing stage 1). This is followed by the involvement of the brainstem, representing stage 2. This was termed a “masked” bidirectional sequence (Kovacs et al., 2018a). This means that pattern 1 described above for subpial ARTAG in non-CBD cases starting at basal areas and proceeding towards lobar subpial location is masked by the predominant end-feet tau immunoreactivity in subpial locations of lobar areas in CBD (Kovacs et al., 2018a). This concept exemplifies that classical ARTAG and its sequential distribution patterns can be present as “comorbid tauopathy” in primary tauopathies characterized by prominent and distinctive astroglial tau pathologies such as TAs or AP. Thus, these overlaps and combinations of astroglial pathologies might influence interpretations of tau immunoreactivities.

## Synthesis: The Pathogenic Relation of Tau and Astroglia

The factor or event that triggers tau aggregation in diverse conditions is yet to be identified. The pathological process of tau aggregation is thought to lead to a *gain of toxic function* paralleled by a loss of physiological functions of tau which alter synaptic functions (Goedert et al., 2017a). Overexpression of mutant human tau leads to filament formation and recapitulates features of human tauopathies (Goedert, 2016). However, overexpression of wild-type human tau in mouse brain does not result in the accumulation of filamentous inclusions in astroglia (Götz et al., 1995), which might question the early role of astroglia in the aggregation process of tau. On the other hand, astroglial activation and reaction seems to precede neuronal loss in PiD (and other FTLD proteinopathies; Kersaitis et al., 2004), supporting an early role of astroglia in the pathogenesis of neurodegeneration itself, eventually before protein aggregation takes place.

Interestingly, divergent patterns of transcriptional associations for neuronal and astroglial tau lesions have been described in PSP (Allen et al., 2018). While neuronal tau pathology is positively associated with a brain co-expression network enriched for synaptic and PSP candidate risk genes, astroglial tau pathology is positively associated with a microglial gene-enriched immune network (Allen et al., 2018). This observation can facilitate the understanding of why there is a difference between brain regions and whether different brain regions preferentially accumulate more neuronal or astroglial tau.

**Figure 3:**
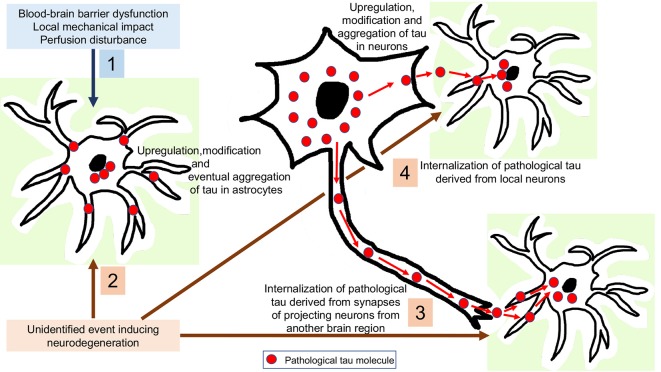
Interpretation of astrocytic tau accumulation. 1: Astroglia expresses subtle levels of tau, which is upregulated and then hyperphosphorylated as a response to a yet unidentified neurodegeneration-inducing event. 2: Tau-overexpression and hyperphosphorylation occurs in astrocytes as a response to a non-neurodegenerative event such as blood-brain barrier dysfunction, perfusion disturbance or a local mechanical impact. 3: Internalization of pathological tau derived from synapses of projecting neurons from another brain region. 4: Internalization of pathological tau derived from local neurons.

To interpret astrocytic tau accumulation the following theoretical possibilities can be considered (Figure 3). First, that astroglia does express subtle (or with current methods is undetectable) levels of tau, which is upregulated and then hyperphosphorylated as a response to an unidentified neurodegeneration-inducing event. There are only scarce studies, which claim that tau might be expressed in astrocytes. For example, positive immunostaining for tau in the normal human brain and astrocytic tumors (Shin et al., 1991; Miyazono et al., 1993) and mRNA expression of tau in astrocytic tumors (Miyazono et al., 1993) have been reported, and the authors considered that astrocytes have a potential to express tau through neoplastic transformation and reactive processes (Miyazono et al., 1993). These observations need to be replicated using current methods, since this would widen the pathophysiological interpretation of astrocytic tau accumulation in NDDs and normal brain aging. However, it is currently more favoured that astrocytes do not contain endogenous tau. Indeed, the pioneering study on tau mRNA expression examining samples of the hippocampus and cerebral cortex evaluated dark-field photomicrographs following hybridization with different probes and found expression only in neurons and not glia (Goedert et al., 1989). In this respect, it is still a puzzle why phosphorylated-tau positive astrocytes are observed in ARTAG without neuronal tau accumulation. This could support the concept of low amounts of tau expression astroglia and suggest that accumulation of tau in astrocytes does happen independently from neuronal tau, but does not necessarily represent a neurodegenerative cause. As exemplified by ARTAG, tau accumulation in astrocytes might reflect the various impacts that individuals can be affected by during life including mechanical impact, perfusion disturbance, or barrier dysfunction.

The most universal view is that astrocytic tau can only be explained by its internalization from the extracellular milieu since astrocytes have been found to express phagocytic receptors such as those phagocytizing synapses (Chung et al., 2013) or axonal mitochondria (Davis et al., 2014) in the brain. Although heparan sulfate proteoglycans have been proposed to be responsible for fibrillary tau uptake in various cell types, astrocytic uptake of tau uptake was found to be an independent process (Perea et al., 2019). Since some brain regions accumulate pathological tau in astroglia before neurons (Ling et al., 2016; Kovacs et al., 2018a), it can be also proposed that these tau-positive astrocytes might phagocytize pathological tau derived from projecting neurons. Another possibility is that astrocytes take up tau secreted from the cytoplasm of local neurons. For these scenarios, presynaptic vesicular secretion, direct translocation from the neuronal cytoplasm across the plasma membrane, release *via* secretory lysosomes, microvesicle shedding, or exosome release could be the source of pathological tau (Sebastián-Serrano et al., 2018) for uptake by astroglia. The observation of an inverse correlation between astroglial and neuronal tau pathology in experimental models was interpreted as support for the transmission of pathological tau from neurons to neighboring astrocytes, or as inter-astrocytic transfer of tau (Narasimhan et al., 2017). Accumulation of tau in astroglia might clear tau from the extracellular space and buffer the neurotoxicity of pathological tau until astrocytes become overloaded. Even without accumulating tau, astroglia in a brain affected by tauopathy might show dysfunction. This is exemplified by an observation indicating that astrocyte transplantation may be neuroprotective in a mouse line transgenic for human P301S tau (Hampton et al., 2010). On the other hand, astroglial tau could be detrimental to neurons. Indeed, neuronal degeneration can be detected in the absence of neuronal tau inclusions but associated with astroglial tau pathology (Forman et al., 2005). Further studies speculated that oxidative damage leads to GFAP fragmentation, indicating dysfunction of pathological tau-harboring protoplasmic astrocytes in PSP, associated with neuronal dysfunction (Santpere and Ferrer, 2009; Song et al., 2009). Studies in cultured hippocampal neurons suggest that extracellular tau oligomers accumulate in astrocytes followed by disrupted intracellular Ca^2+^ signaling and Ca^2+^-dependent release of gliotransmitters altogether contributing to synaptic dysfunction (Piacentini et al., 2017). In summary, the neuroprotective function of astroglia, in particular the proper protection of neurons from glutamate neurotoxicity, may be impaired early in tauopathies and/or astroglia may gain novel neurotoxic properties (Sidoryk-Wegrzynowicz and Struzyńska, 2019). The latter might be linked to neuroinflammatory responses also, in particular that astroglia may upregulate genes encoding pro-inflammatory mediators and that microglial activation may induce the formation of a subtype of astrocytes (A1; Liddelow et al., 2017) with potential detrimental effects on neurons (Sidoryk-Wegrzynowicz and Struzyńska, 2019).

In AD, evaluation of the astroglial response is complicated by the fact that in addition to tau, Aβ is also present and this might lead to different reactions as in primary tauopathies or ARTAG. Indeed, astrocytes may directly participate in APP metabolism and Aβ clearance (Ries and Sastre, 2016) but they seem to be implicated in neurofibrillary degeneration of AD where the number of activated astrocytes correlates not only with the burden of tangles (Serrano-Pozo et al., 2011) but with the stage of tangle formation (i.e., pretangles to ghost tangles) formation (Sheng et al., 1997). Ultrastructural examination of ghost tangles revealed the presence of astrocytic processes with paired-helical filaments suggesting their incorporation by astrocytes (Ikeda et al., 1992). On the other hand, a population-based study suggested that classical Alzheimer-type pathology is a poor explanatory variable for the astrocyte response seen in the aging brain and rather that astrocytes may respond to other age-associated events (Simpson et al., 2010). Thus, the astroglial response in the neurodegenerating or aging brain might not exactly reflect the pathogenesis of primary tauopathy-related astroglia response where tau protein itself accumulates in these cells.

## Questions to be Answered

Astroglia accumulate pathological tau in various disease conditions and normal brain aging. The question remains whether astrocytic tau accumulation uniformly precedes, parallels, or follows neuronal tau in all situations. It also remains unknown whether and how astroglial tau accumulation can develop independently from neuronal tau (see for example subpial TSAs). There is a paucity of data on whether different populations of astrocytes are vulnerable in different disorders accumulating tau in astrocytes and where astrocytic tau originates from, including the mechanism of secretion of tau taken up potentially by astrocytes. There is currently a lack of data on whether astrocytes have a peculiar biochemical tau signatures that differs from neurons. Finally, it remains to be determined whether tau neuroimaging or bodily fluid tau-biomarkers represent astroglial tau accumulation or purely neuronal tau accumulation. Thus, the role of astroglia in tau-associated diseases has still unexplored fields that require communication between glia researchers and tau experts.

## Author Contributions

GK wrote the article.

## Conflict of Interest

The author declares that the research was conducted in the absence of any commercial or financial relationships that could be construed as a potential conflict of interest.
